# Identification of cis-regulatory modules encoding temporal dynamics during development

**DOI:** 10.1186/1471-2164-15-534

**Published:** 2014-06-27

**Authors:** Delphine Potier, Denis Seyres, Céline Guichard, Magali Iche-Torres, Stein Aerts, Carl Herrmann, Laurent Perrin

**Affiliations:** INSERM, UMR1090 TAGC, Marseille, F-13288 France; Aix-Marseille Université, UMR1090 TAGC, Marseille, F-13288 France; CNRS, Marseille, France; Laboratory of Computational Biology, Center for Human Genetics, University of Leuven, Herestraat 49, P.O. Box 602, 3000 Leuven, Belgium; IPMB, div. of Theoretical Bioinformatics, University of Heidelberg and German Cancer Research Center, Im Neuenheimer Feld 280, Heidelberg, 69120 Germany

**Keywords:** Cis-regulatory modules, Temporal control, Motif discovery, Transcription, Drosophila metamorphosis, Cardiogenesis

## Abstract

**Background:**

Developmental transcriptional regulatory networks are circuits of transcription factors (TFs) and cis-acting DNA elements (Cis Regulatory Modules, CRMs) that dynamically control expression of downstream genes. Comprehensive knowledge of these networks is an essential step towards our understanding of developmental processes. However, this knowledge is mostly based on genome-wide mapping of transcription factor binding sites, and therefore requires prior knowledge regarding the TFs involved in the network.

**Results:**

Focusing on how temporal control of gene expression is integrated within a developmental network, we applied an *in silico* approach to discover regulatory motifs and CRMs of co-expressed genes, with no prior knowledge about the involved TFs. Our aim was to identify regulatory motifs and potential trans-acting factors which regulate the temporal expression of co-expressed gene sets during a particular process of organogenesis, namely adult heart formation in Drosophila. Starting from whole genome tissue specific expression dynamics, we used an *in silico* method, cisTargetX, to predict TF binding motifs and CRMs. Potential Nuclear Receptor (NR) binding motifs were predicted to control the temporal expression profile of a gene set with increased expression levels during mid metamorphosis. The predicted CRMs and NR motifs were validated *in vivo* by reporter gene essays. In addition, we provide evidence that three NRs modulate CRM activity and behave as temporal regulators of target enhancers.

**Conclusions:**

Our approach was successful in identifying CRMs and potential TFs acting on the temporal regulation of target genes. In addition, our results suggest a modular architecture of the regulatory machinery, in which the temporal and spatial regulation can be uncoupled and encoded by distinct CRMs.

**Electronic supplementary material:**

The online version of this article (doi:10.1186/1471-2164-15-534) contains supplementary material, which is available to authorized users.

## Background

Embryonic development is regulated by extensive transcriptional networks that drive cell-specific patterns of gene expression [1, 2]. At a molecular level, transcriptional programs are orchestrated by the recruitment of transcription factors (TFs) to enhancer elements or *cis*-regulatory modules (CRMs). CRMs act as modular units that integrate inputs from multiple TFs giving rise to a specific spatio-temporal output of gene expression [3]. Developmental gene regulatory networks (GRNs) are circuits of transcription factors and *cis*-acting DNA elements that control expression of downstream regulatory and effectors genes. Understanding how the underlying *cis*-regulatory networks produce temporal and spatial gene expression is an essential step towards deciphering metazoan development.

Constructing such networks requires identification of the regulatory genes involved and the characterization of their temporal and spatial expression patterns. Identification of downstream genes and associated CRMs, and mapping TF binding sites within them is a prerequisite. Two approaches are classically used to identify target genes and CRMs. The first is based on chromatin immuno-precipitation (ChIP) using an antibody against a particular TF, followed by next generation sequencing (ChIP-seq). This approach, combined with the computational analysis of conserved binding sites, has been successful in identifying target genes and improving our knowledge of involved GRNs in a number of studies. For example, in Drosophila, it has allowed exhaustive identification of CRMs and target genes of TFs involved in mesoderm specification and diversification [4–7]. The second approach (sometimes used in combination with ChIP-seq) is based on genetic perturbation of identified TFs and large scale analysis of expression level changes. This allows identification of developmental programs and their downstream genes, such as those associated with myoblast diversification [8]. A similar strategy, in which the proneural transcription factor Atonal was over-expressed, lead to the identification of 204 Atonal target genes [9]. However, both of these approaches require pre-existing knowledge regarding the TFs involved. In cases in which such knowledge is lacking, when searching for TFs beyond those already identified, *in silico* approaches often fail to identify relevant signals in metazoan genomes. Given the large size of the non-coding genome, approaches based on motif-discovery often fail to distinguish signal from background noise. Reducing the search space by focusing on proximal regions may help uncover some signal, albeit at the expense of deliberately ignoring large, potentially functional regions [10, 11].

Others have investigated alternative strategies, based on machine-learning approaches applied to training sets of validated enhancers. These studies have extracted sequence features from a set of CRMs driving similar expression, which were used to build a model that was applied genome-wide to predict further CRM candidates [12, 13]. These are “TF-blind” approaches in that they do not require pre-existing knowledge of the relevant transcription factors, and the CRM search can be carried out without restriction to particular regions. However, they depend crucially on the availability of a sufficiently large set of homogeneous, experimentally validated CRMs showing similar spatio-temporal activity, which is not always available.

In this study, we applied an alternative strategy, focusing on how temporal control of gene expression is integrated within a gene regulatory network. Our aim was to identify CRMs (and their associated potential transcription factor binding sites (TFBSs)), responsible for a strict temporal expression profile of sets of co-expressed genes during adult heart formation in Drosophila. Starting from whole genome tissue specific expression dynamics, a recently developed *in silico* method, *cis*TargetX [9, 14] was used to predict TF binding motifs and CRMs. It combines genome wide motif cluster predictions with gene set enrichment analysis. Because *cis*TargetX uses a large sequence space (5 kb upstream from the transcription start site and all introns) and a large motif collection (1981 position weight matrices) to predict potential regulatory motifs, we reasoned that it could allow prediction of regulatory motifs without prior definition of the potential TFs.

Adult heart formation in Drosophila occurs by complete remodeling of the larval organ during metamorphosis. This process is cell autonomously controlled by the ecdysone Receptors (EcRs). All aspects of heart remodeling are dependent on the activity of these nuclear receptors (NRs), which, in particular, modulates expression and activity of Hox genes [15]. We previously investigated the sequence of events that occur at the transcriptional level during the remodeling process. A precise molecular portrait of adult heart formation was drawn through whole genome analysis of the temporal dynamics of heart-specific gene expression [16]. This led to the description of clusters of genes expressed in the cardiac tube with strict temporal expression patterns. This study highlighted the involvement of a handful of conserved signaling pathways, each being involved in specific aspects of cardiac remodeling. It further supported the central role played by ecdysone signaling. Indeed, as observed in other tissues, the cardiac specific transcriptome dynamics revealed the sequential activation of ecdysone responsive genes, which are known downstream effectors of activated EcRs. A number of these ecdysone response genes are themselves nuclear receptors that constitute a transcriptional cascade and may drive the dynamics of cardiac remodeling. Starting from this biological system, our goal was to evaluate the feasibility of predicting transcriptional control modalities at the temporal level, without prior knowledge of the CRMs and TFs involved.

To predict potential transcription factor motifs involved in the regulatory process, we used *cis*TargetX, a computational approach described previously [9]. This tool uses a comprehensive library of 1981 position weight matrices, combined with phylogenetic conservation, to identify potential cis-regulatory modules common to a cluster of co-expressed genes. It produces high confidence target predictions from statistical correlations between the input, a co-expressed gene set and the background, genome wide target prioritization [9]. Recently, we successfully used *cis*TargetX to predict a regulatory network at play during cardiac aging [17]. Here we used *cis*TargetX to predict motifs for TFs involved in the temporal control of gene expression during heart metamorphosis, and to predict associated CRMs.

We focused on one particular set of co-expressed genes whose expression is initiated late during remodeling, at 42 hours after puparium formation. To our knowledge, no master regulators have been described that control this specific up-regulation of gene expression at this time point. Potential regulatory motifs and CRMs were predicted for this gene set based on their evolutionary conservation, and over-representation in the surrounding non-coding sequences of co-expressed genes with a high statistical significance. Next, we performed *in vivo* validations of predicted CRMs, and demonstrated that the tested CRMs reproduce the expected temporal expression pattern. In addition, we demonstrated that this temporal expression pattern is abolished when the motifs are mutated. These motifs resemble the nuclear receptor family motifs. We further demonstrate that several nuclear receptors, namely Hr39, Eip75B and Hr46, displaying dynamic expression during metamorphosis, are essential for the temporal pattern of the enhancer-reporter. Hence, our approach was successful in identifying CRMs and potential TFs regulating the temporal activation of the target genes. In addition, our results suggest a modular architecture of the regulatory machinery, in which the temporal and spatial regulations are distinct.

## Results

### Regulatory motifs and target gene discovery within spatio-temporally co-expressed gene sets using conserved binding sites and gene ranking

**Figure 1:**
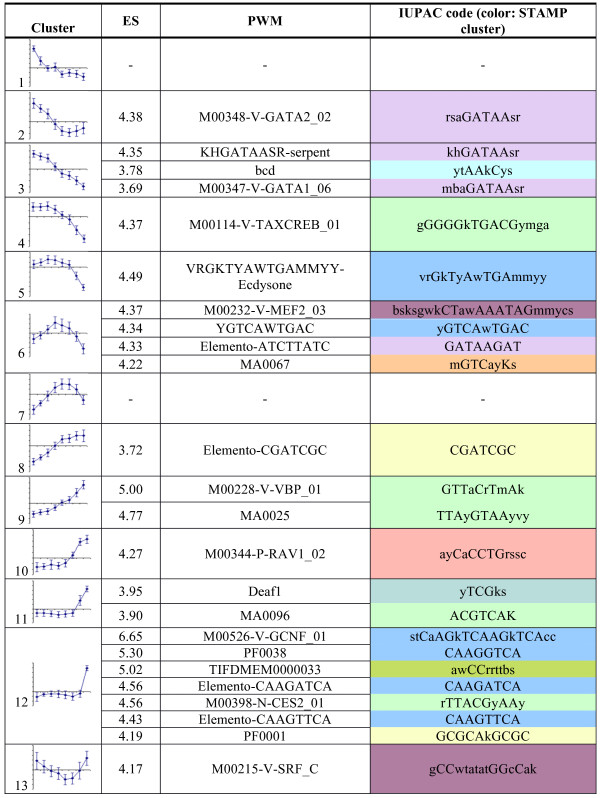
***In silico*** **predictions of transcriptional regulatory motifs within gene sets defined from cardiac remodelling transcriptome dynamics.** PWM matrices logos are provided in Additional file 1: Figure S1. The temporal expression pattern of the 13 co-expressed gene sets are shown (as defined in [16]; y axis: expression level, x axis: time). Cardiac expression dynamics was analyzed at 21, 24, 27, 30, 33, 36, 42 and 48 hours APF. The PWMs that rank corresponding gene sets above the automatic enrichment score (ES) threshold are displayed, together with their enrichment score and IUPAC sequence (PWMs converted to IUPAC sequence with RSATools “convert matrix” using Drosophila melanogaster as background model [18]). Color code corresponds to stamp clustering of all retrieved PWM across all clusters. Blue: Nuclear receptor R type motifs; green: bZIP motifs; purple: Mef2 like motifs; Pale purple: GATA-like motifs. Note that no PWMs were recovered above the automatic threshold in cluster 1 and 7.

By using a high sampling of time points from 21 to 48 hours after puparium formation (APF), a precise picture of the transcriptome landscape of adult heart formation could be described [16]. In this experiment, 1660 genes showed significant levels of differential expression throughout the time-course. Self-organizing map (SOM) clustering of these dynamically expressed genes demonstrated temporal and progressive gene expression changes, across 13 distinct clusters, with diverse patterns of temporal changes, including continuous up-regulation, continuous down-regulation, an early peak of expression, a late peak of expression, and more complex temporal patterns (Figure 1).

To identify potential transcriptional regulatory motifs and associated target genes from these gene sets, we choose to use the *cis*TargetX method, recently published [9, 14]. Statistical over-representation of motifs in the non-coding DNA around genes was calculated for all clusters of temporally co-expressed genes (Figure 1 and Additional file 1: Figure S1). Some analyzed gene sets were well ranked for motifs expected to be bound by known components of the cardiac GRN. This is the case for GATA-like position weight matrices (PWMs) recovered for clusters 2, 3 and 6 and of the Mef2 related PWMs in clusters 6 (M00232-V-mef2) and 13 (M00215-SRF). Pnr/GATA4 and Mef2 have central roles in the cardiac GRN (see [19] for review) and the score observed here may suggest that they have a role in the regulation of corresponding gene sets. Several matrices for basic region leucine zipper (bZIP) transcription factors were also significantly well ranked for several gene sets (Figure 1 and Additional file 1: Figure S1), which may point to a potential role of a bZIP TF in adult cardiac GRN. As a matter of fact, two bZIP TFs (Vrille (Vri) and Pdp1) are dynamically expressed during cardiac tube remodeling (respectively in cluster 4 and 11, see [16]). Of particular interest were PWMs representing the DNA binding specificity of NRs that were recovered in several clusters, namely clusters 5 (VRGKTYAWTGAMMYY-Ecdysone), 6 (YGTCAWTGAC, closely related to ecdysone Receptors (EcRs) matrices) and 12 (M00526-V-GCNF_01, binding specificity of the vertebrate NR GCNF). Indeed, NRs are known temporal regulators of Drosophila development [20] and the good ranking observed, suggested that they play a role in temporal regulation of corresponding gene sets. Indeed, we previously demonstrated that EcR is cell autonomously required for heart remodeling and that its cardiac specific loss of function fully prevents adult heart formation [15]. Hence, the recovery of an EcR-type motif in cluster 6 which corresponds to genes induced early during the remodeling process may be viewed as a validation of our approach.

**Figure 2:**
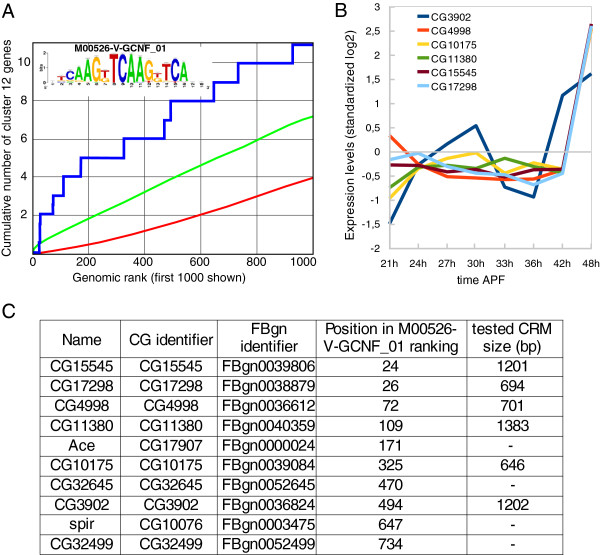
**Enrichment of the nuclear receptor – type GCNF motifs in cluster 12 gene set. A)** ROC curve showing significant enrichment in putative NR binding sites (GCNF position weight matrix) among the 42 genes constituting cluster 12 (y axis) compared to a randomized set of 1000 Drosophila genes (x axis) using cisTargetX. The blue curve shows the detection of cluster 12 genes, the red line a random distribution, and the green curve shows a 2 sigma interval from random. **B)** Expression profile of the 10 best ranked genes. All genes display marked expression increase after 42 h after puparium formation (APF). **C)** The 10 genes are listed together with the size of the 6 tested CRMs (see Additional file 1: Table S2 and Additional file 1: Figure S2 for details).

The best enrichment score across all clusters was found in cluster 12 for the NR motif M00526-V-GCNF_01. In addition, genes in cluster 12 are well ranked for a number of other NR PWMs (Figure 1 and Additional file 1: Table S1), supporting a central role for NR motifs in the regulation of associated genes. To test this hypothesis and validate our approach, we decided to investigate the potential role of corresponding motifs *in vivo*. Among the 10 best ranked genes from cluster 12, we selected genomic fragments that contain high-scoring clusters of the GNCF-like type matrix motif in the vicinity of six genes (Figure 2) and manually extended the fragments on both sides, retaining flanking sequences with high *phastCons* [21] conservation scores across 12 Drosophila genomes to prevent potential CRM fragmentation (Additional file 1: Figure S2). Six putative CRMs of genes from cluster 12 were predicted in this way, with sizes ranging from 646 bp to 1383 bp (Figure 2 and Additional file 1: Table S2).

### Predicted CRMs reproduce the temporal expression pattern of associated genes

**Figure 3:**
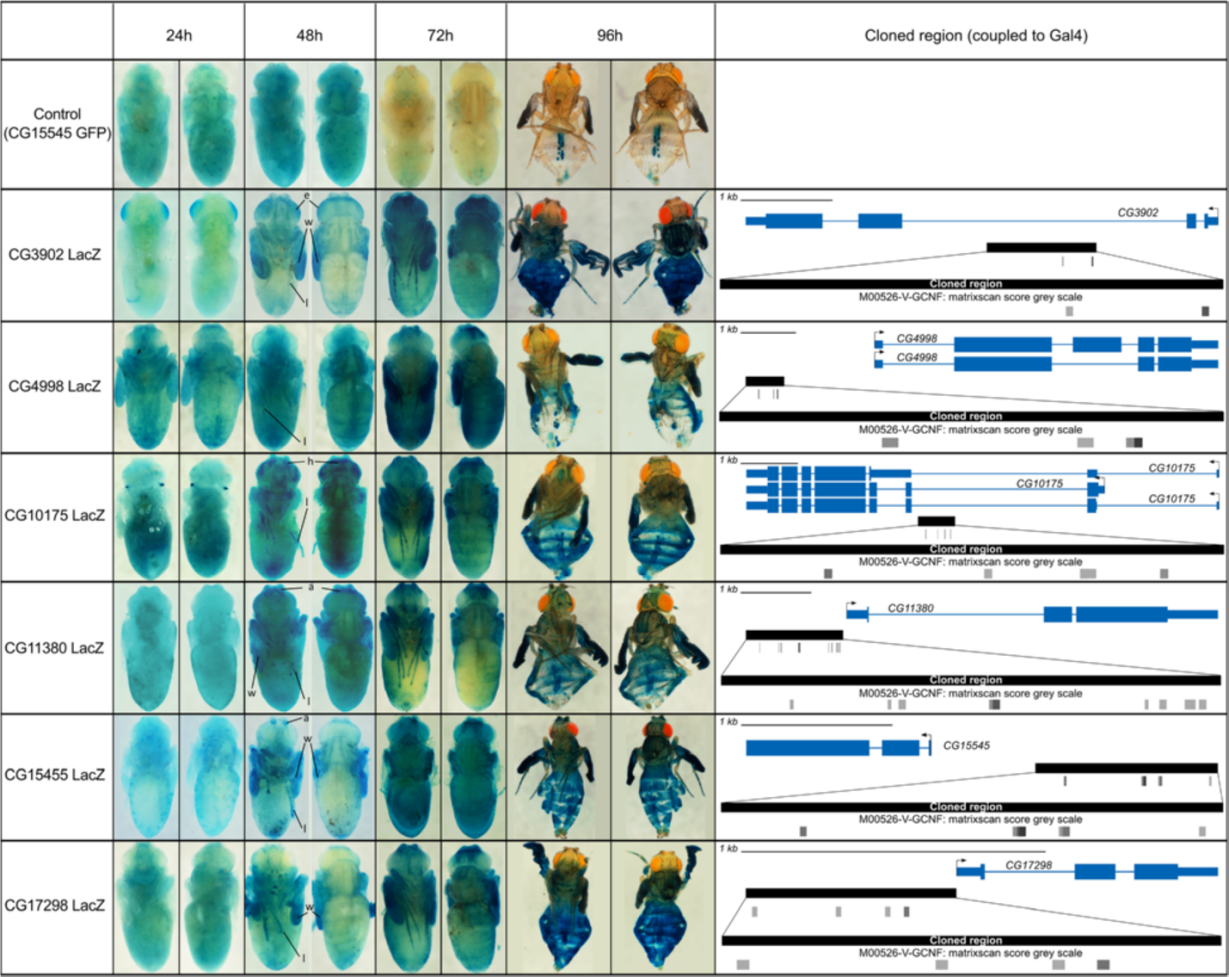
**Gene reporter assays reveal the temporal dynamics driven by tested CRM.** Right: Each CRM predicted by *cis*TargetX is schematically represented (see Additional file 1: Figure S2 for a detailed description). Vertical black lines represent evolutionarily conserved GCNF clusters as predicted by cisTargetX. Horizontal boxes summarize regions tested by transgenic assays. Left: Reporter activity was examined at different times after puparium formation (APF, indicated on top). A ventral and a dorsal view are shown in each case. Top: CG15545 was also analyzed using a GFP reporter (Figure 4) which serves as a control for βGal staining. No βGal expression is visible, except on pharate adults (96 h APF) in pericardiac cells, which indicate endogenous βGal activity in this tissue. **24 h APF**: No βGal activity was observed, except in a few discrete tissues in CG3902-LacZ and CG10175-LacZ flies (respectively in the developing eye and in discrete spots at the basis of the head). **48 h APF**: In all LacZ transgenic lines, reporter construct induce βGal expression in a variety of tissues (weak staining was occasionally observed at 42 h, not shown). a: antennae, e: eye, h: head, l: legs, m: muscles, w: wings. **72 h APF**: Maximum βGal activity was observed around this time point. **96 h APF**: X-Gal staining in pharate adults is due to stability of βGal, since no expression at this stage was observed on GFP-reporter constructs (see Figure 4).

The six predicted CRMs were tested using reporter gene assays in transgenic Drosophila pupae for *in vivo* validation, using Lac-Z as a reporter gene. Remarkably, all six tested constructs showed β - Galactosidase (βGal) expression in a temporal pattern comparable to their predicted associated genes. Indeed, similarly to genes in cluster 12 which show increased expression at 48 h APF, all six tested CRMs activate βGal expression between 24 h APF and 48 h APF in a variety of tissues (Figure 3). In addition, examination of their expression dynamics at later pupal stages shows further increase in expression after 48 h with maximum βGal activity around 72 h APF (Figure 3).

**Figure 4:**
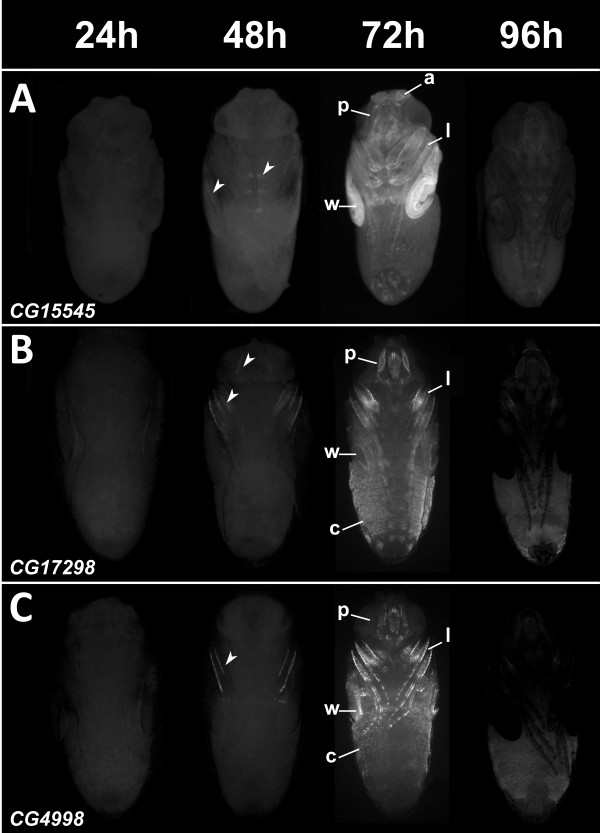
***In vivo*** **GFP reporter activities of predicted NR target enhancers.** Enhancer GFP reporter assays for CG15545 **(A)** CG17298 **(B)** and CG4998 **(C)** at increasing time points during metamorphosis. Top: timing of pupal development in hours APF (After Puparium Formation). Expression driven by all 3 enhancers start to be detected at 48 h APF (arrow heads) and their activity increases up to 72 h APF. At 96 h APF, GFP signal is almost not detected. All CRMs drive expression in specific regions of the w: wings, l: legs, a: arista, p: proboscis and in different c: cuticle part.

To facilitate reporter gene expression analysis during metamorphosis, and to increase the spatial and temporal characterization of the reporter expression pattern, we analyzed a subset using a fluorescent reporter expression assay (Figure 4). Although each CRM displays a specific spatial expression pattern with expression in diverse tissues such as various cuticle parts, the wings, legs and different structures in the head, all displayed identical temporal profiles, with reporter activation starting at 48 h APF and peaking at 72 h APF. This expression profile fits very well with the cardiac temporal expression dynamics we started from. Almost all reporter constructs drove expression in all, or a subset of the forming adult wings (Figures 3 and 4). Hence, to confirm the temporal expression profile of genes in cluster 12 and reporter construct in this tissue, green fluorescent protein (GFP) expression was monitored in precisely staged pupal wings of CG15545-GFP flies by quantitative reverse transcription polymerase chain reaction (qRT-PCR) at different time points. A marked increase in expression of GFP was seen between 30 and 48 h APF and between 48 and 72 h APF, as with all endogenous genes examined (Additional file 1: Figure S3). Therefore, CRMs reproduce the temporal pattern of gene expression, and a subset of its spatial pattern. Surprisingly however none of the tested regions drive expression of reporter genes (neither LacZ nor GFP) in the heart. This suggests that in this tissue, spatial and temporal cis-regulatory inputs may be seperate, and that the CRMs with spatial information remain to be discovered (see Discussion).

### NR motifs are required for accurate CRM activity

**Figure 5:**
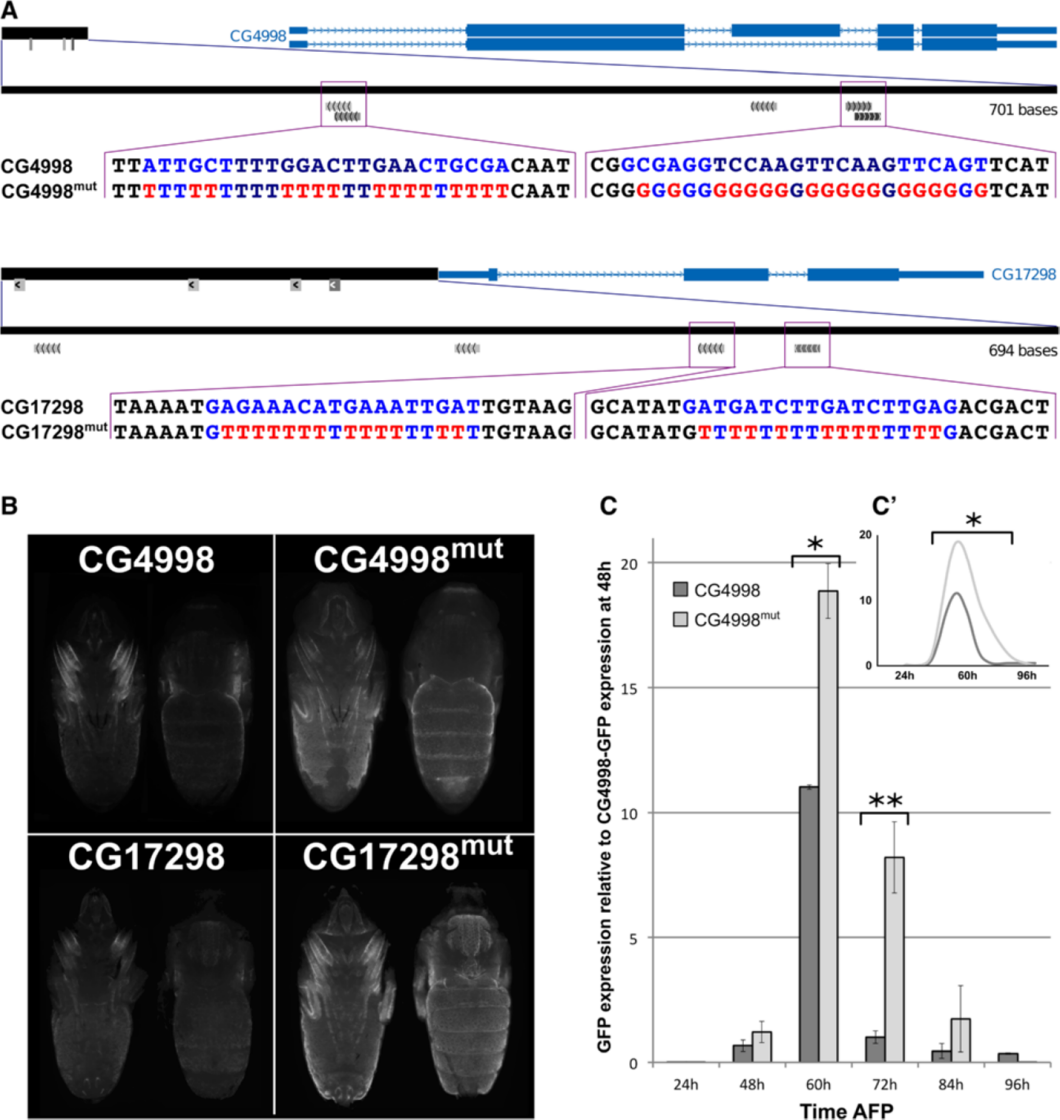
**NR motifs are functional within CRMsNR motifs are functional within CRMs. A)** Schematic representation of mutations performed in NR motifs of CG4998 (top) and CG7298 (bottom) CRMs. The location of CRMs (black rectangles) with respect to corresponding genes is indicated on top, and putative NR motifs are represented as grey boxes. NR motifs sequences are indicated below, together with the corresponding mutated sequences. Note that in CG4998-CRM both putative motifs are comprised of two inverted overlapping motifs. Motifs in light grey are of lesser quality and were not analyzed in this study. **B)** GFP expression pattern at 72 h APF in WT (left) and mutated (right) individuals for CG4998 (top) and CG17298 (bottom) CRMs. A ventral and a dorsal view are shown in each case. NR motif mutations lead to a marked increase of GFP signal. **C)** Time course of GFP expression between 24 and 96 h APF driven by CG4998 and CG4998-mutated CRMs analyzed by qRT-PCR. GFP expression was normalized to RP49 expression levels and expression ratio relative to GFP expression at 48 h in non-mutated CRM are represented (*p < 0.05; **p < 0.001, Student t-test). **C’)** The dynamics of GFP expression in the mutated CG4998 CRM is significantly different from the one observed with wild type CRM (*p < 0.05 χ2 test).

We next investigated whether the predicted nuclear receptor binding sites are necessary for CRM activity by mutating the predicted binding motifs for NR in two enhancers, CG17298 and CG4998 (Figure 5A). In each case, the CRMs display two putative NR motifs, and both were mutated simultaneously. Importantly, as shown in Figure 5B, these mutations strongly increased the GFP signal indicating that the NR motifs represent functional elements within the tested enhancers. Note, that the general de-repression of reporter expression is observed without affecting the spatial pattern. Detailed temporal expression pattern was further analyzed for CG4998-CRM (wild type (WT) and mutated) by qRT-PCR (Figure 5C). Dynamic expression of reporter RNA confirmed the temporal expression pattern observed at the protein level *in vivo*, and demonstrated a peak expression at 60 h APF. Following mutation of the NR motifs, GFP expression was detected at higher levels at all time points analyzed between 48 and 96 h APF.

In conclusion, our *in silico* predictions – which were centered on temporal modalities of gene expression - successfully predicted the cis-regulatory inputs responsible for the temporal expression profile of associated genes and suggest an important role for Nuclear Receptors in this process.

### Nuclear receptors modulate CRM activity with a balanced activation and repression on their target enhancers

The central role played by GNCF-like motifs in the activity of the tested CRMs suggests that one or several NRs may regulate these CRMs and their associated target genes. Indeed, the GCNF PWM that is significantly well ranked among genes from cluster 12 is closely related to several PWMs for Drosophila NRs (Additional file 1: Figure S4). Of note, two of these PWMs are also significantly enriched in the cluster 12 gene set, albeit to a lesser extent compared to M00526-V-GCNF_01 (Additional file 1: Table S1). This moderate enrichment might be attributed to the quality of the respective PWMs. Actually, while M00526-V-GCNF_01 is constituted by a tandem repeat, fitting with the known hetero- or homo-dimerization of NRs at their target sequences [20], the Drosophila NR PWMs that are available are in most cases constituted by a single motif, suggesting that these PWMs only partially reflect the DNA binding properties of corresponding active NRs.

**Figure 6:**
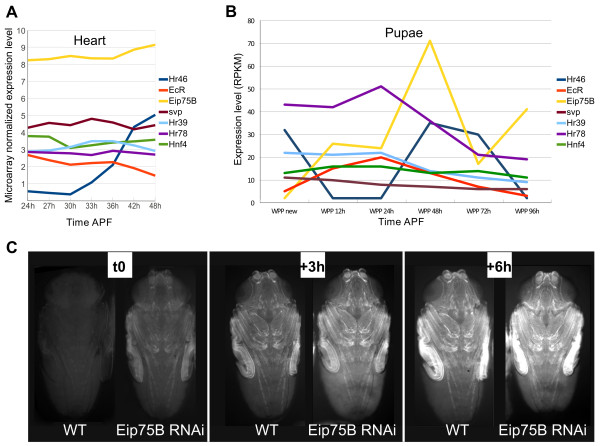
***Eip75B*** **impacts on CG15545-GFP expression. A)** Expression dynamics of NR in the cardiac tube during its remodeling from Zeitouni et al. 2007 [16] time course microarray data. Only those whose expression level is above the detection limit at at least one time point were considered. Expression values after within-array and between-array normalization are plotted. **B)** Expression of these NRs in whole pupae according to flybase modENCODE_mRNA-Seq_U data (http://flybase.org/reports/FBlc0000085.html). Note the particular feature of *Eip75B* and *Hr46* expression which peak at 48 h APF. **C)** Effect of *Eip75B* ubiquitous knock down on CG15545-GFP dynamic expression. White prepupae were selected and grown at 25°C (permissive temperature) for 25 hours and shifted at 29°C (restrictive temperature) for 18 h (t0) to 24 h (t + 6 h). Left: expression of GFP in Tub-Gal4, Gal80ts; CG15545-GFP control individuals (WT). Right: expression of GFP in UAS > *Eip75B*RNAi Tub-Gal4, Gal80ts; CG15545-GFP individuals. *Eip75B* knock down induces a precocious activation of CG15545-GFP expression noticeable at t0 and lead to increased GFP expression at t3 and t6. Representative individuals. All (30) animals examined displayed this precocious activation of GFP (see Material and Methods).

NRs are present across all eukaryotes and possess at least one zinc finger-C4 domain involved in DNA binding and a helical domain involved in hormone binding [20]. In Drosophila, 21 NRs have been identified of which 7 are expressed during heart remodeling (Figure 6A). Since we started from a gene set dynamically expressed in this tissue, we focused on these NRs even though none of the CRMs tested drove expression in this tissue. All of these 7 NRs share extensive similarities in their DNA binding affinity with the vertebrate GCNF (Additional file 1: Figure S4) and all are expressed at moderate to high levels during metamorphosis in whole individuals (Figure 6B). We therefore tested whether they influence the CG15545-CRM driving GFP expression by manipulating their activity specifically during metamorphosis using the TARGET system [22]. To obtain optimal pupal development while modulating NR activity before the onset of CRM expression, pupae were allowed to develop at 25°C up to 25 h APF and were then moved to 29°C to induce Gal4 activity. The expression profiles of *Hr46* and *Eip75B* in whole flies was particularly suggestive since they both have peak expression at 48 h APF, precisely at the onset of these CRMs activity, these NRs were therefore tested first. Even when using the TARGET system and this relatively late temperature shift, *Hr46* knock down using RNAi hampered pupal development (data not shown), thus preventing the analysis of its loss of function effects on CRM activity at mid pupal stages. However, *Hr46* overexpression induced a precocious activation of CG15545-GFP reporter expression. Indeed, the expression dynamics of the CG15545-GFP reporter started at earlier stages of pupal development when *Hr46* was overexpressed (Additional file 1: Figure S5). This indicates that Hr46 behaves as an activator of CRM activity. In addition, *Hr46* ectopic expression at the larval stage in the central nervous system, induced ectopic expression of CG15545-CRM in a subset of neurons (Additional file 1: Figure S5B) while no GFP expression is observed in the CG15545-GFP reporter line at larval stages in a WT context. This confirms the activating potential of Hr46 on this CRM. The potential lack of corresponding Hr46 ligand or co-factor at this stage may explain the low number of neurons that respond to Hr46 in these conditions. We next investigated the potential regulatory effect of Eip75B. *Eip75B* knockdown was achieved through RNAi expression during pupal stages with the TARGET system. As for *Hr46* gain of function, using the temporal shift described above - first 25 h of pupal development at 25°C, the permissive temperature for gal80ts, and then shifting to 29°C - did not affect the timing of pupal development. Remarkably however *Eip75B* loss of function induces a clear precocious activation of CG15545-GFP expression (Figure 6C), thus pointing to a repressive role of Eip75B upon the activity of this CRM.

Other expressed NRs were also tested for their activity with respect to CG15545-GFP expression. However, *EcR* inactivation during metamorphosis blocks pupal development [15] and was therefore not tested. *Hnf4* and *Hr78* RNAi mediated knockdown had no effect on CG15545-GFP expression dynamics (not shown). On the contrary, *Hr39* knockdown accelerated CG15545-GFP expression dynamics (Additional file 1: Figure S5C) very much like *Eip75B*, indicating that both genes behave as negative regulators of the temporal pattern.

In conclusion, both their expression and the genetic manipulations described here point to a central role of *Hr39* and *Eip75B* in the temporal repression of the CRMs identified in this study. Given that putative NR binding motifs are functional within tested CRMs and are involved in their repression, *Hr39* and/or *Eip75B* may well be directly involved in their temporal expression control. *Eip75B* encodes three protein isoforms designated E75A, E75B, and E75C [23]. Interestingly it has been recently demonstrated that E75A directly represses transcription of EcR target genes by opposing to EcR/Usp binding on enhancers [24]. It is therefore conceivable that *Eip75B* directly repress the CRMs tested here.

At metamorphosis, ecdysone, through *EcR* activation, induces a cascade of transcriptional activation involving a number of NRs that are intricately coordinated. The observed effect of Hr46 on CRM activity could therefore be due to an indirect effect on *Eip75B* and *Hr39* expression. Alternatively, several reports have established that *Eip75B* gene products can form a complex with Hr46 which switches Hr46 activating potential into a repressive one (see for example [25]). Since *Hr46* and *Eip75B* are induced at 48 hours APF both in the heart, and the whole organism, their products may indeed dimerize to form a repressive complex that controls the expression of the CRMs. In this case, the activating potential of Hr46 reported above may be due to a lack of *Eip75B* gene expression in neurons, and to a titration of *Eip75B* gene products by *Hr46* overexpressed at metamorphosis.

## Discussion

Starting from co-expressed gene sets and without prior knowledge of the TFs involved in their spatio-temporal transcriptional regulation, our analysis shows that we were able to identify the correct motif from a set of co-expressed genes and accurately predict target genes of individual TFs. The unbiased approach of cisTargetX, based on the enrichment of a comprehensive collection of TF motifs, allows discovery of relevant motifs without restricting the analysis to a particular class of transcription factors. The robustness of the approach is confirmed by the fact that (i) several motifs corresponding to nuclear receptors are found to be enriched for the transient cluster analyzed here, and (ii) applying this method to other clusters identifies further motifs corresponding to nuclear receptors, corresponding well to the fact that these clusters must have strict temporal control. A striking result is the fact that the motifs discovered here appear to form homotypic clusters. While it is not possible to rule out the fact that an un-characterized TFBS might also be present, the dense arrangement of NR binding sites is reminiscent of the homotypic CRMs involved in early embryogenesis which respond to spatial morphogen gradients. It is therefore tempting to hypothesize a mechanism by which the clusters of NRs respond to a temporal gradient of nuclear receptors. Hence, this organization of binding sites might represent a more general feature during development, but not limited to early Drosophila embryogenesis [26].

### Dissecting spatial and temporal cis-regulatory inputs

Since regulatory motifs and CRMs were predicted and compared for gene sets that have distinct temporal patterns but common spatial expression, our objectives were specifically directed towards predicting regulatory inputs that achieve temporal expression pattern. Indeed, while we started from genes co-expressed in the cardiac tube, none of the tested CRMs drove detectable expression in cardiac myocytes, indicating that our predictions did not retrieve the heart specific spatial information. This failure to reproduce cardiac expression pattern indicates that for the genes analyzed, the cis-regulatory modules that drive spatial expression in the cardiac tube might be distinct from, and located at a distance from the temporal CRMs. In an attempt to predict the regulatory sequences driving spatial expression in the cardiac tube, we have used cisTargetX with all 1660 dynamically expressed genes during adult heart formation. The common property of this gene set is its spatial expression in the heart. Therefore, using this gene set, cisTargetX might allow predicting regulatory motifs responsible for this spatial expression pattern. Interestingly, a number of PWMs known to be bound by bZIP transcription factors were significantly enriched in this gene set (not shown), suggesting that one or several bZIP transcription factor(s) may play a role in the expression pattern of these genes. Of note, among the few CRMs known to drive spatial expression in the cardiac tube, one, the cardiac specific Tin enhancer (TinC) was shown to have functional bZIP motifs [27]. In addition, a recent study has underlined the important role of the bZIP TF E4BP4 in embryonic heart development in vertebrates [28], thus supporting a potential function of one or several bZIP TF(s) in the cardiac GRN. Future work aimed at deciphering the cardiac function of bZIP transcription factors, and of their potential cognate enhancers should allow some light to be shed on the modalities of cardiac restricted spatial expression at metamorphosis and its relatedness to temporal control of gene expression.

The identified regulatory regions nevertheless display characteristic spatial expression patterns, for instance in the developing pupal wings and legs. This most probably indicates that in these tissues, the spatial control of gene expression is driven by TFs that bind in the vicinity of the motifs responsible for temporal control. Alternatively, one could hypothesize that NRs themselves are responsible for the spatial expression pattern of the CRMs. However a function of NRs in the spatial control of genes and CRM expression is not supported by our experimental data. Indeed, mutations of NR binding motifs only affect the timing of expression, and have no effect on spatial control of reporter gene expression. The same is true regarding the phenotypes induced by NR gain and loss of function at pupal stages. Furthermore all tested NRs are expressed in the heart (this was our selection criteria) but none of the analysed CRMs are expressed there, indicating that the spatial expression pattern of NRs cannot explain the spatial expression pattern of the CRMs.

### Integrating temporal control within GRNs

Developmental timing mechanisms are intricately linked to pattern formation, and disruption of temporal programs can cause organism wide changes in development that can result in catastrophic birth defects [29]. At the level of GRNs, this implies that robust mechanisms ensure precise coordination of both spatial and temporal transcriptional control. Some of these mechanisms are “encoded” in the structure of the network themselves. For instance, network motifs such as feed-forward loops confer dynamics to the networks and ensure defining the temporal order of specification events [30]. There are other mechanisms which also drive time-directed developmental processes. One such mechanism concerns the molecular oscillators that govern the vertebrate segmentation clock. In this case however, the nature of the clock pacemaker still remains elusive [31]. Another mechanism -based on the temporal control of TF activity- was recently pointed to, in a well-documented example of terminal differentiation in Drosophila. Indeed, Kondo et al. [32] demonstrated that a small peptide (pri) triggers amino terminal truncation of the svb protein, switching its activity from a full-length repressor to a cleaved activator, providing temporal control to the GRN of epidermal differentiation. A study of heterochronic genes in C. elegans -which ensure that stage specific developmental programs occur in the appropriate sequence- demonstrated that miRNAs play a central role in the temporal control of gene expression during larval development [33]. While the transcriptional regulation of these miRNA encoding genes remain elusive, recent studies indicated that a central transcriptional regulator of heterochronic genes is the nuclear receptor DAF-12 [34]. Moreover, a number of studies in different model organisms and humans identified NRs as major regulators of developmental timing, usually as targets of hormonal cues. For instance, NRs are involved in humans to trigger the marked changes that occur during puberty and adolescence. In Drosophila, pulses of the steroid hormone ecdysone, which bind to, and activate EcR, control a number of developmental transitions during embryonic and post-embryonic development. In particular, three ecdysone pulses activate genetic regulatory hierarchies that coordinate the developmental changes associated with Drosophila metamorphosis [35]. Ecdysone pulses trigger the progression of the pupae into different stages through the transcription of a particular cascade of genes, most of which are themselves nuclear receptors. We previously demonstrated that EcR function is required for all aspects of cardiac tube remodeling, thus indicating that it is located at the top of adult heart formation GRN [15]. We now show that NR motifs are central in the temporal regulation of CRMs of genes activated late during the remodeling process. In addition, we provide evidence suggesting that NRs might be direct trans-regulators of these CRMs, thus suggesting that they are part of the cardiac GRN during metamorphosis, acting downstream of EcR function to control the expression of these late genes. Other NR PWMs were recovered using *cis*TargetX on different co-expressed gene sets, suggesting that other NRs may play similar roles at earlier stages of cardiac remodeling.

Alternative modalities of temporal control of gene expression have been reported in Drosophila metamorphosis. In particular, it has recently been shown that *Eip93F*, one of the primary targets of the ecdysone receptors that encodes a non-nuclear receptor transcription factor, plays a central role in the timely restricted expression of *distalless* (and most probably of many other genes) during adult morphogenesis [36]. *Eip93F* is expressed at high levels during the early steps of heart remodeling [16] and it is therefore possible that it participates in the temporal control of corresponding gene sets. The current lack of knowledge about its DNA binding specificity however prevents challenging its involvement in the process using our approach. Another study implicated the importance of core promoters’ choice in the temporal control of gene expression during metamorphosis of the wing [37]. Although it was not in the focus of our study, it is possible that in the heart also the type of core promoters’ usage may play an additional role in the temporal control of gene expression during adult heart formation.

## Conclusions

Using an *in silico* strategy, and without any prior knowledge regarding the involved TFs, our approach identified CRMs and potential TFs acting on the temporal regulation of a co-expressed gene set. Indeed, based on evolutionary conservation and over-representation in the surrounding non-coding sequences of co-expressed genes, potential regulatory motifs and CRMs were predicted and were both validated *in vivo*. We further demonstrate that several nuclear receptors, displaying dynamic expression during the biological process analyzed, are essential for the temporal pattern of the enhancer-reporter; the fact that some act as activators while others are repressors suggests a subtle balance between these opposite effects, as is the case for the spatial expression during early embryogenesis. Therefore, our strategy was successful at identifying CRMs and TFs involved in the temporal dynamics of gene expression. In addition, our results suggest a modular architecture of the regulatory machinery, in which the temporal and spatial regulations can be uncoupled and encoded by distinct CRMs.

## Methods

### Dataset

The gene expression dynamics during heart remodeling from 21 h to 48 h After Puparium Formation (8 time points) was described previously [16]. In this experiment, we identified 2394 genes that exhibited significant differential expression between time-points in using modified *t*-statistic significance analysis of microarrays (SAM) [38] with estimated *q*-values (false discovery rates) of ≤ 0.05. Among them, 1660 genes showed significant levels of differential expression at least 1.8-fold in at least one condition through our time-course analysis. Those 1660 genes were clustered in 13 expression profiles using SOM clustering. All 13 clusters were independently submitted for *cis*TargetX analysis.

### Motif over representation analysis in the 13 clusters

*cis*TargetX was used with the following parameters: - Assembly & Scoring: “dm2 (April 2004) with Cluster-Buster”; - motif collection: “Used in Aerts & al. PLoS Biology 2010 (1981 PWM)”; Z-score thresold: “Determine threshold automatically”; receiver operating characteristic (ROC) threshold for area under the curve (AUC) calculation: “0.03”; Genomic threshold for visualization: “1000”. The search space used is 5 kb upstream of all transcripts TSS (Transcription Start Site) and their introns. If a neighboring gene (or a host gene exon in case of an intronically hosted gene) is less than 5 kb away, the search space is reduced and truncated at this neighboring gene (or exon) boundary. In the case of cluster 12, in order to inspect further PWM, cisTargetX was run a second time with the previous parameters except for the Z-score threshold, which was decreased to 2.5.

### PWM clustering

In order to visualize similar PWM (given the redundancy in the PWM library), we used STAMP [39] with default parameters and the “-chp” option to obtain clusters of similar PWM. A color code was used to visualize PWM belonging to related families.

### Determination of drosophila TFs predicted to bind GCNF motif

As GCNF is a motif representing the binding site of a vertebrate NRs, we selected the 22 Drosophila TFs belonging to the Nuclear hormone receptor family in the UniProt database (Hr39, EcR, tll, usp, Hr46, Hr96, Hr78, Hr4, Hr38, ftz-f1, svp, kni, Eip75B, eg, Eip78C, knrl, Hnf4, usp, Hr51, ERR, dsf, Hr83) with the following request, family: “nuclear hormone receptor family” AND organism: “7227”. We collected PWMs representing NRs binding sites present in the *cis*TargetX library (1981 PWMs) or in the Fly Factor Survey database and use STAMP with default options to build a similarity tree (Additional file 1: Figure S4).

### CRM selection and delimitation

To delineate the putative CRMs, we selected DNA fragments around clusters of GNCF TFBS in the vicinity of the top ranked genes. Fragments were manually extended on each side in order to retain conserved flanking sequences with high *phastCons* conservation scores in the UCSC genome browser (“conservation” track in the “Comparative Genomics” section). All tested fragments are presented as UCSC genome browser screenshots in Additional file 1: Figure S3.

### Molecular cloning and transgenesis

All fragments described in Figure 2 were amplified starting from W^1118^; cantonS genomic DNA and cloned by standard molecular cloning techniques using the gateway system in three different reporter vectors; a GFP reporter (pH-attB-Dest, [9]), a LacZ reporter vector produced by replacing eGFP by LacZ in the “pH-attB-Dest” vector from and a gal4 reporter (SMG4-Gal4 generous gift from Nicolas Gompel). The intermediate cloning vector used is the pDONR221. Vectors were introduced by electroporation in DH5α competent cells to be amplified. We performed mutations for two binding sites predicted for GCNF motif in CG4998 (attgcttttggacttgaactgcga to tttttttttttttttttttttttt and gcgaggtccaagttcaagttcagt to gggggggggggggggggggggggg) and in CG17298 (gatgatcttgatcttgag to tttttttttttttttttg and gagaaacatgaaattgat to cccccccccccccccccc). Long polymerase chain reaction (PCR) primers containing the mutations were used and the mutated fragments were cloned in the SMG4-gal4 vector.

To avoid position dependent expression variability, we used targeted PhiC31 transgenesis [40]. All constructs were inserted at P2(3 L)68A4 landing site using PhiC31 integrase. Lines containing the SMG4-Gal vector were crossed with a UAS_GFP line.

### RNA extraction and qRT-PCR

For qRT-PCR on dissected pupal wings, dissected wings from four CG15545-GFP individuals were used for each biological replicate and three biological replicates were analyzed. Staged pupae were dissected under stereo microscope and dissected wings immediately placed in Trizol® (Life Technologies) on ice and RNA extraction (see bellow) was performed extemporaneously.

For the analysis of GFP expression on CG4998 and CG4998* -CRMs, total RNAs was extracted from 5 individuals per replicate and 3 biological replicates were analyzed. Staged pupae of corresponding genotypes were collected, ground in Trizol® and RNA extraction (see below) was performed extemporaneously. A list of primer pairs used for qRT-PCR analysis is provided bellow.

Total RNA was extracted from samples in triplicate using Trizol® according to standard procedures. The integrity of RNA samples was assessed using Agilent 2100 Bioanalyzer and RNA Nano CHIP kit (Agilent). Total RNA concentration was measured using a NanoDrop ND-1000 Spectrophotometer (ThermoScientific) and its purity was evaluated by absorbance ratios, 260/280 and 260/230. cDNA was synthesized from 500 ng of DNase I (Promega)-treated total RNA in a 25 μl reaction volume using qScript cDNA SuperMix (Quanta Biosciences) according to supplier instructions. cDNA samples were diluted five-fold for real-time qRT-PCR reactions. Gene-specific transcription levels were determined in a 15 μl reaction volume in triplicate using SYBR Green (Invitrogen) and a BioRad CFX (Biorad) for pupal wings; and a Stratagene MX3000P real-time qPCR system (Agilent) for whole pupae total RNA analysis following the manufacturer’s instructions. Standard cDNA samples with 4-fold serial dilutions were used for PCR efficiency calculations. Amplifications were performed as follows: for pupal wings, 8 min at 95°C, 40 cycles of 10s at 95°C/30s at 60°C and for pupae total RNA, 5 min30 at 95°C, 40 cycles of 15 s at 95°C/1 min at 60°C. To verify the specificity of amplicons, a melting curve analysis was carried out from 60°C to 95°C. Real-time qRT-PCR reactions of the standard, test cDNA samples and no template controls using the same primer set were analyzed together in the same 96-deep well plate (Agilent or BioRad) in order to minimize run-to-run variations and use exactly the same threshold setting (user defined baseline subtracted curve fit) for determination of the threshold cycle values (Ct). Parallel samples were processed using the same batch of reagents to minimize overall sample-to-sample variations. Gene specific primers are listed in Additional file 1: Table S3.

### Data analyses

Data analyses were manually performed to calculate the key variables including PCR efficiency (E(%) = (10exp[-1/slope]-1)×100) and squared correlation coefficients (R^2^) of primer sets and expression ratios of target genes were normalized according to expression levels of RP49. In Figure 5, GFP expression values are displayed relative to GFP expression in the CG4998-GFP reporter line at 48 h. In Additional file 1: Figure S3, expression values are displayed relative to expression at 30 hrs. In addition, a logarithmic scale of the fold change was used for better display. To determine significance of the results, statistic comparisons were performed with a (one sample test or paired test) Student’s t-test. Statistical comparisons of dynamic temporal curve (Figure 5C’) were performed with a chi-Square test.

### βGalactosidase staining

24 h, 48 h, 72 h and 96 h APF individuals were extracted from their pupal cage. They were fixed using a PBS 1×, formaldehyde 3,7% and triton 0.01% solution and then washed with Phosphate-Buffered Tween (PBT) three times and with Phosphate-Buffered Saline (PBS) once. The pupae were then sonicated to perforate their membrane in order to allow a X-Gal staining (10/15 min in X-gal buffer: PSB 1×; KferriCN 4 mM; KferroCN 4 mM; MgCl2 2 mM; NP40 1%; X-gal 0,04 mg/ml).

### GFP expression analysis

For GFP expression analysis, a batch of 10 flies per conditions and genotypes were analyzed in triplicate and representative individuals are shown. Images were acquired using a high-resolution video camera (CoolSNAP HQ Monochrome, Roper Scientific, Inc), mounted on a Zeiss Stereo Lumar V12 binocular microscope with a Neolumar 0.83 objective. Image acquisition was performed with the Metamorph/Metafluor software (universal Imaging, West Chester, PA). Individuals shown in Figures 4, 5 and 6 were dissected out their pupal case and mounted in Voltaleff oil 3S prior imaging.

### Drosophila strains and fly husbandry

The following stocks were obtained from the Bloomington Drosophila Stock Centre. UAS-mcd8-GFP, UAS-dsRNA > *Eip75B,* UAS-dsRNA > *Hr39,* UAS-dsRNA > *Hfn4,* UAS-dsRNA > *Hr78,* P(tub-GAL80[ts]), P(tub-GAL4). UAS > *Hr46*; UAS-dsRNA > *Hr46* and the elav > GeneSwithGal4 were obtained from V Monnier (Paris).

### Timing of pupal development and control of Gal4 induction

Onset of pupal development corresponds to white pupae that were selected on the basis of spiracle eversion, absence of reaction following forceps contact and absence of tanning. Individuals were kept for further development in an air incubator at 25°C. Tub(Gal4); P(tub-GAL80[ts]) and CG15545CRM > GFP transgenes were combined in the same lines and crossed with appropriate UAS lines. Crosses with wild type flies served as controls. Development was allowed to proceed at 22°C until white pupal stage and individuals were then shifted to 25°C for 25 hours and to restrictive temperature (29°C) for the indicated period of time. Except for Hr46 and EcR whose knockdown hampered pupal development, none of the analysed NR affected the timing of pupal development when knockdown was accomplished following this time schedule. Therefore the effect seen after Eip75B and Hr39 RNAi mediated loss of function cannot be attributed to a precocious pupal development.

For GeneSwitch experiments, second instar larvae were shifted to regular food medium containing either 0 μg/ml (control) or 100 μg/ml of RU486 (Mifepristone, Sigma) and GFP expression pattern was analyzed at third instar larval stage.

## Electronic supplementary material

Additional file 1: Table S1: CisTargetX analysis of cluster 12 gene set with 2.5 Z score cutoff. **Table S2.** Detailed genomic coordinates of tested CRMs. **Table S3.** List of primer pairs used for Q-RT PCR analysis. **Figure S1.** Logos of PWM outlined in Figure 1. **Figure S2.** Details of tested CRM.**Figure S3.** qRT-PCR in developing pupal wings of CG15545-GFP individuals. **Figure S4.** Sequence based comparison of NR motifs. **Figure S5.** Hr46 and Hr39 regulate CG15547-GFP expression. (DOCX 6 MB)

## Abbreviations

NR: Nuclear receptor
GRN: Gene regulatory network
CRM: Cis regulatory module
PWM: Position weight matrice
TF: Transcription factor
TFBS: Transcription factor binding site
bZIP: Basic region leucine ZIPper
EcR: Ecdysone receptor
ChIP: Chromatin immuno-Precipitation
APF: After puparium formation
SOM: Self-organizing map
ROC: Receiver operating characteristic
AUC: Area under the curve
PCR: Polymerase chain reaction
qRT-PCR: Quantitative reverse transcription polymerase chain reaction
WT: Wild type
GFP: Green fluorescent protein
TSS: Transcription start site
SAM: Significance analysis of microarrays.
