# Supplementary material for: Identification of cis-regulatory modules encoding temporal dynamics during development
Source: BMC Genomics. 2014 Jun 27;15(1):534. doi: 10.1186/1471-2164-15-534 (PMC4097164; doi:10.1186/1471-2164-15-534)

**Additional file 1 (Tables S1, S2, S3 and Figures S1, S2, S3, S4, S5)**

**Table S1**. **CistargetX analysis of cluster 12 gene set with 2.5 Z score cutoff**. Motifs in bold correspond to those above the automatic threshold cutoff. Underlined motifs correspond to matrices for *Drosophila* NR.

| Motif | Enrichment score |
| --- | --- |
| M00526-V-GCNF_01 | **6.65** |
| PF0038 | **5.31** |
| TIFDMEM0000033 | **5.02** |
| CAAGATCA | **4.57** |
| M00398-N-CES2_01 | **4.57** |
| CAAGTTCA | **4.43** |
| PF0001 | **4.19** |
| ACTTGAC | 3.88 |
| AACCGGTT | 3.83 |
| M00033-V-P300_01 | 3.62 |
| *YSAAGGWCRCHRM-ftz* | *3.58* |
| CTGCGCA | 3.55 |
| CAAGGTC | 3.52 |
| CATCAAGG | 3.45 |
| M00045-V-E4BP4_01 | 3.32 |
| *Hr46* | *3.32* |
| M00229-N-SKN1_01 | 3.25 |
| SelexConsensus_Kr | 3.20 |
| M00109-V-CEBPB_01 | 3.12 |
| PF0153 | 3.11 |
| TGATCTC | 3.06 |
| M00117-V-CEBPB_02 | 3.04 |
| PF0108 | 2.98 |
| M00359-P-BZIP911_02 | 2.93 |
| M00340-V-ETS2_B | 2.77 |
| M00511-V-ERR1_Q2 | 2.76 |
| M00771-V-ETS_Q4 | 2.76 |
| M00040-V-CREBP1_01 | 2.76 |
| PF0025 | 2.74 |
| M00016-I-E74A_01 | 2.73 |
| TTGACAC | 2.73 |
| MA0089 | 2.69 |
| TIFDMEM0000085 | 2.64 |
| M00260-V-HLF_01 | 2.64 |
| M00237-V-AHRARNT_02 | 2.60 |
| ACACCTG | 2.60 |
| CCTTGCG | 2.59 |
| PF0154 | 2.58 |
| M00218-P-MYBPH3_01 | 2.56 |
| TGGCGCC | 2.55 |
| M00039-V-CREB_01 | 2.55 |

**Table S2: Detailed genomic coordinates of tested CRMs**

| Name | tested CRM size (bp) | Coordinates (dm3) | | |
| --- | --- | --- | --- | --- |
| CG15545 | 1201 | chr3R | 26698680 | 26699880 |
| CG17298 | 694 | chr3R | 17108225 | 17108918 |
| CG4998 | 701 | chr3L | 16329762 | 16330462 |
| CG11380 | 1383 | chrX | 1084009 | 1085391 |
| CG10175 | 646 | chr3R | 19351434 | 19352079 |
| CG3902 | 1202 | chr3L | 18888856 | 18890057 |

**Table S3: List of primer pairs used for Q-RT PCR analysis**.

| Primer Name | Forward sequence | Reverse Sequence |
| --- | --- | --- |
| RP49 | GACGCTTCAAGGGACAGTATCTG | AAACGCGGTTCTGCATGAG |
| eGPF | CCATGCCGAGAGTGATC | GAAGCGCGATCACATGG |
| mGFP | TGGAAGCGTTCAACTAGAAG | CCCAGCAGCTGTTACAAACTC |
| CG4998 | TGTCAACCACGATGTGGAGT | AGACAGGCGGGACTAATGTG |
| CG3902 | CTACCGTGGCATTACCACCT | AGGTTCCCAGGATGTTCTCC |
| CG10175 | TCCGAGGATCCTATGCTGAC | GTGAGGGTGAGTGTGTGTGG |
| CG15545 | CAGGAGAGTCCTCCGTTCAG | CCAACATCCTCTCCAGATCC |

**Figure S1. Logos of PWM outlined in Table 1.** Gene clusters in which the PWMs are enriched are defined on the left. The color code represented is the same as in Table 1 and shows the PWM clustering by STAMP.

**
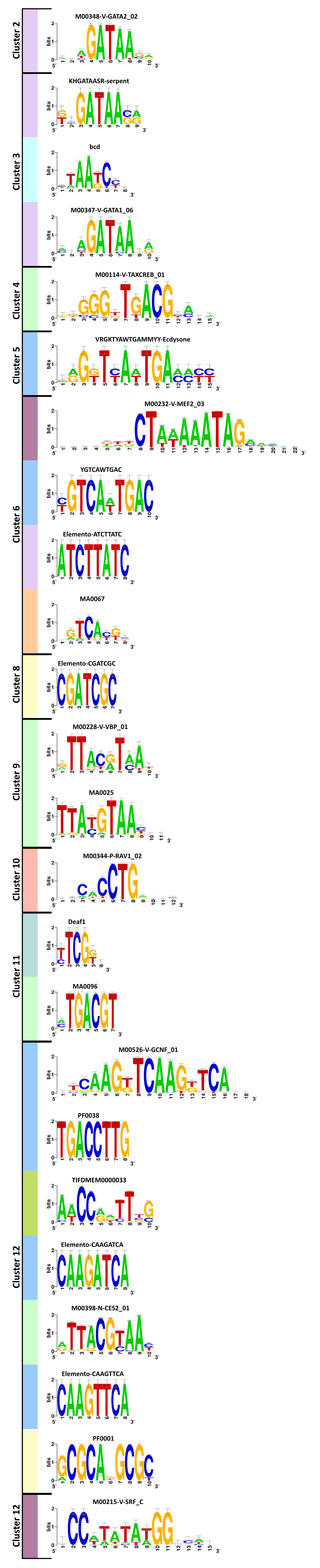
**

**Figure S2**. **Details of tested CRM.** UCSC genome browser view encompassing genomic regions around the 6 tested CRMs. Four tracks are displayed: "cloned region" track represents the genomic regions cloned - "M00526-V-GCNF_matrixscan" track represents NR predicted binding sites scores obtained with M00526 transfac PWM using matrixscan. A grey scale is used to represent the scores (the darker, the higher) - "Flybase Protein-Coding Genes" track is representing coding exons by a thick blue line, UTRs by medium blue line, introns by a thin blue line and the arrows indicates the transcriptional direction - The last track represent phastcons conservation scores across 12 Drosophila species and 3 insects, black peaks indicating high phastcons conservation score.


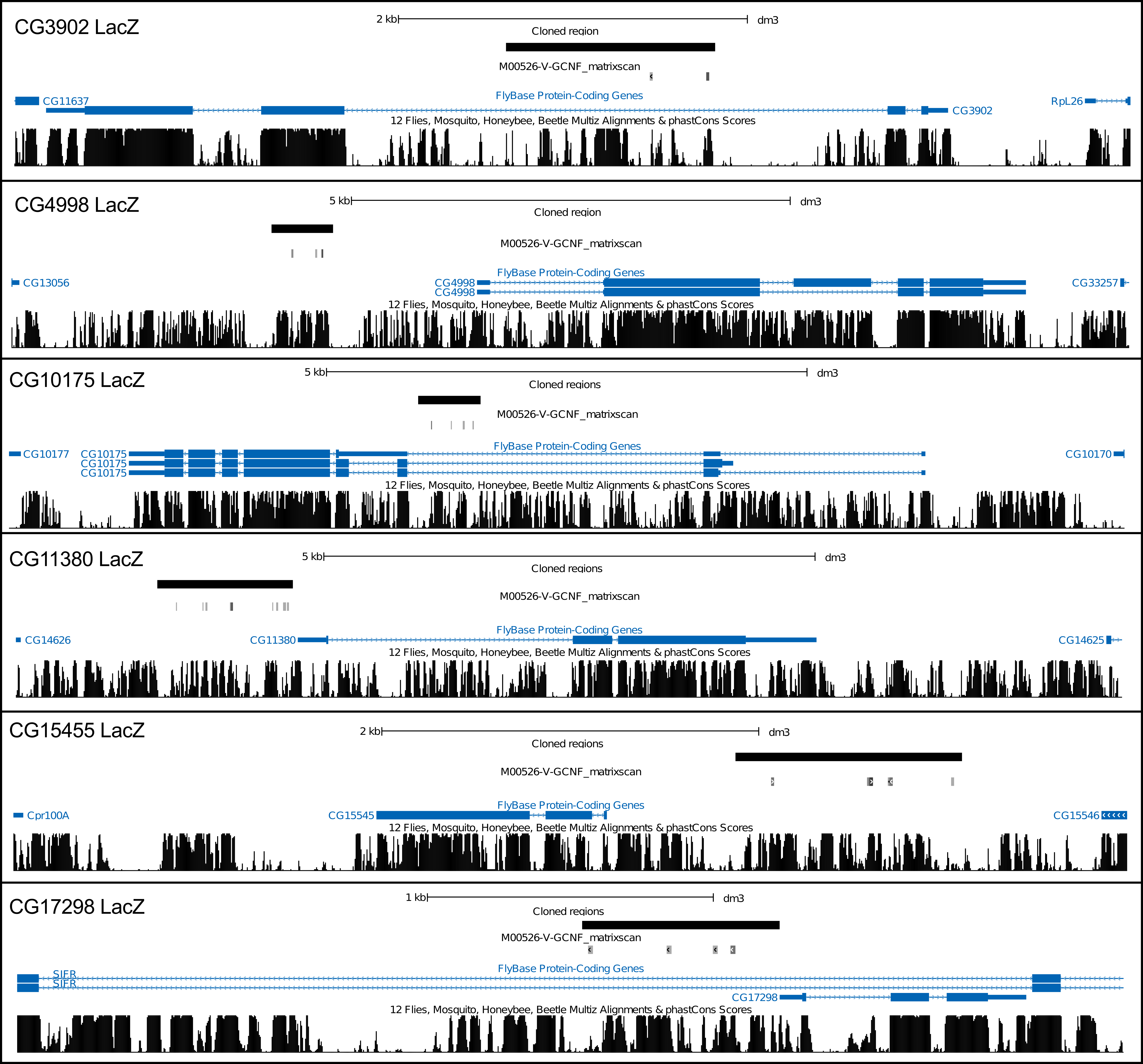


**Figure S3**. **qRT-PCR in developing pupal wings of CG15545-GFP individuals**. CG3902, CG4998, CG15545, CG10175 and eGFP expression levels, first normalized by RP49 expression, are shown as log2 of their fold change at 48 and 72 h APF compared to 30 h APF. All tested genes display marked increased expression at 48 and 72 hrs APF. Note that the GFP expression in the transgenic line shows activation in the wings at 48h APF as the endogenous CG15545 gene.


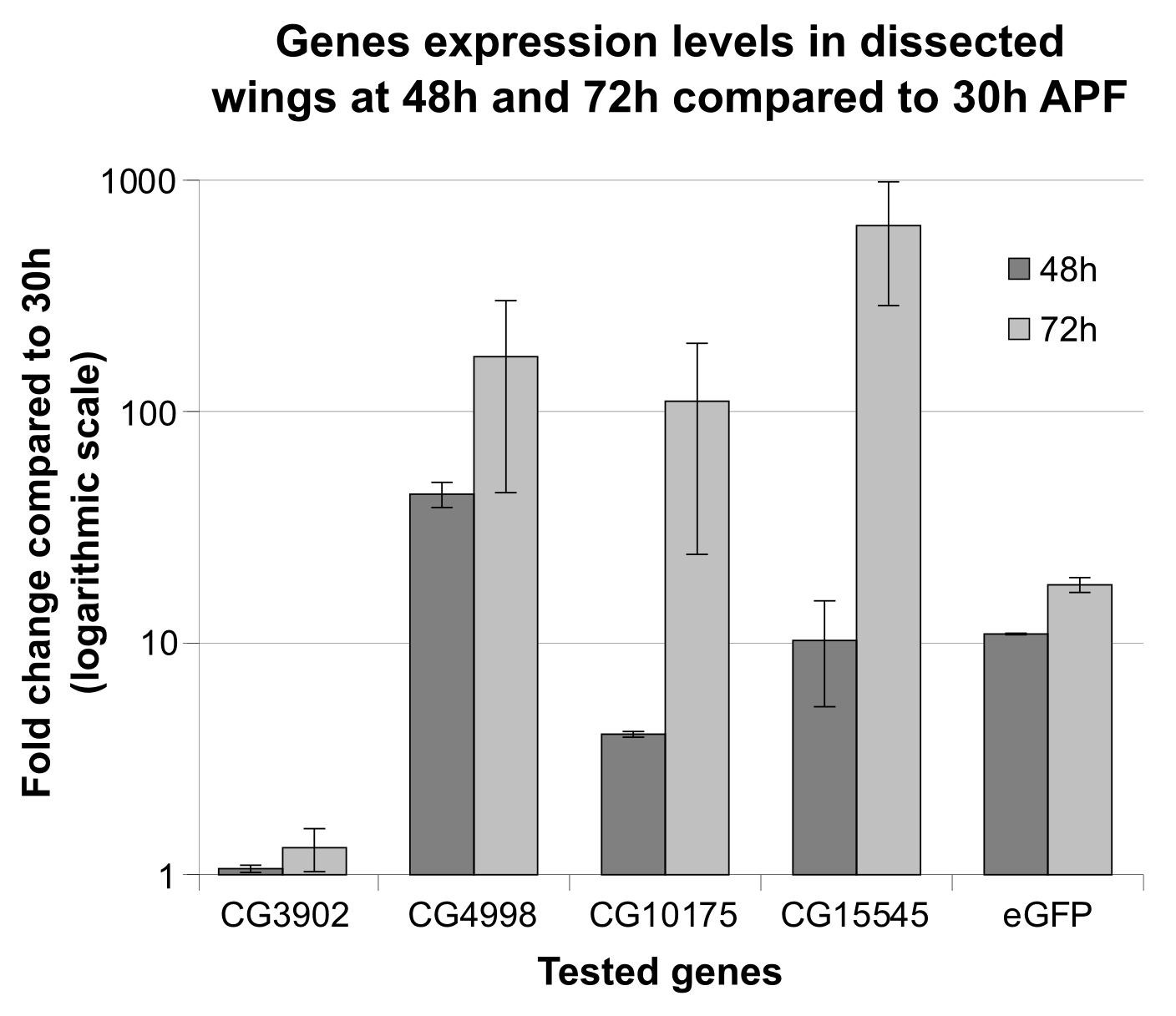


**Figure S4**. **Sequence based comparison of NR motifs**. Tree representing the similarity between the GCNF motif ranked in cluster 12 (figure 1) and all available Drosophila NR motifs calculated using STAMP (see materials and methods). At the top of the tree, the familial NR binding profile (based on multiple alignments of all NR motifs) is also provided, showing a common core.





**Figure S5.** **Hr46 and Hr39 regulate CG15547-GFP expression.** A) Effect of Hr46 ubiquitous overexpression on CG15545-GFP expression dynamics. Representative individuals are shown. Top: expression dynamics of GFP in Tub-Gal4, Gal80ts; CG15545-GFP control individuals. Bottom: expression dynamics of GFP in UAS>Hr46; Tub-Gal4, Gal80ts; CG15545-GFP individuals. Hr46 overexpression induces a precocious activation of CG15545-GFP expression noticeable at t0 and t2 in all (30) individuals analyzed. White prepupae were selected and grown at 25°C for 25 hours and shifted to 29°C for 18 (t0) to 24 h (t+6).

B) Hr46 was ectopically expressed in the nervous system at larval stage using the inducible elav-GeneSwitchGal4 (elav-GSGal4). (1/2): anterior and posterior most segments of 3rd instar larvae (elav-GSGal4; 15545-GFP) individuals grown on RU486-free food. (1’/2’): anterior and posterior most segments of 3rd instar larvae (same genotype: elav-GSGal4; 15545-GFP) grown on RU486 containing food. RU486 mediated activation of GeneSwitchGal4 in larval nervous system induces ectopic GFP expression in a subset of neurons (arrow heads). Such ectopic expression was observed in 80% of the larvae analyzed (n=20) while none of the control (-RU) larvae displayed such expression pattern.

C) Effect of Hr39 ubiquitous knock down on CG15545-GFP dynamic expression. White prepupae were selected and grown at 25°C (permissive temperature) for 25 hours and shifted to 29°C (restrictive temperature) for 18h (t0) to 24 h (t+6h) . Representative individuals are shown. Left: expression of GFP in Tub-Gal4, Gal80ts; CG15545-GFP control individuals (WT). Right: expression of GFP in UAS>Hr39RNAi Tub-Gal4, Gal80ts; CG15545-GFP individuals. In all (30) analyzed individuals, Hr39 knock down induces a precocious activation of CG15545-GFP expression noticeable at t2.


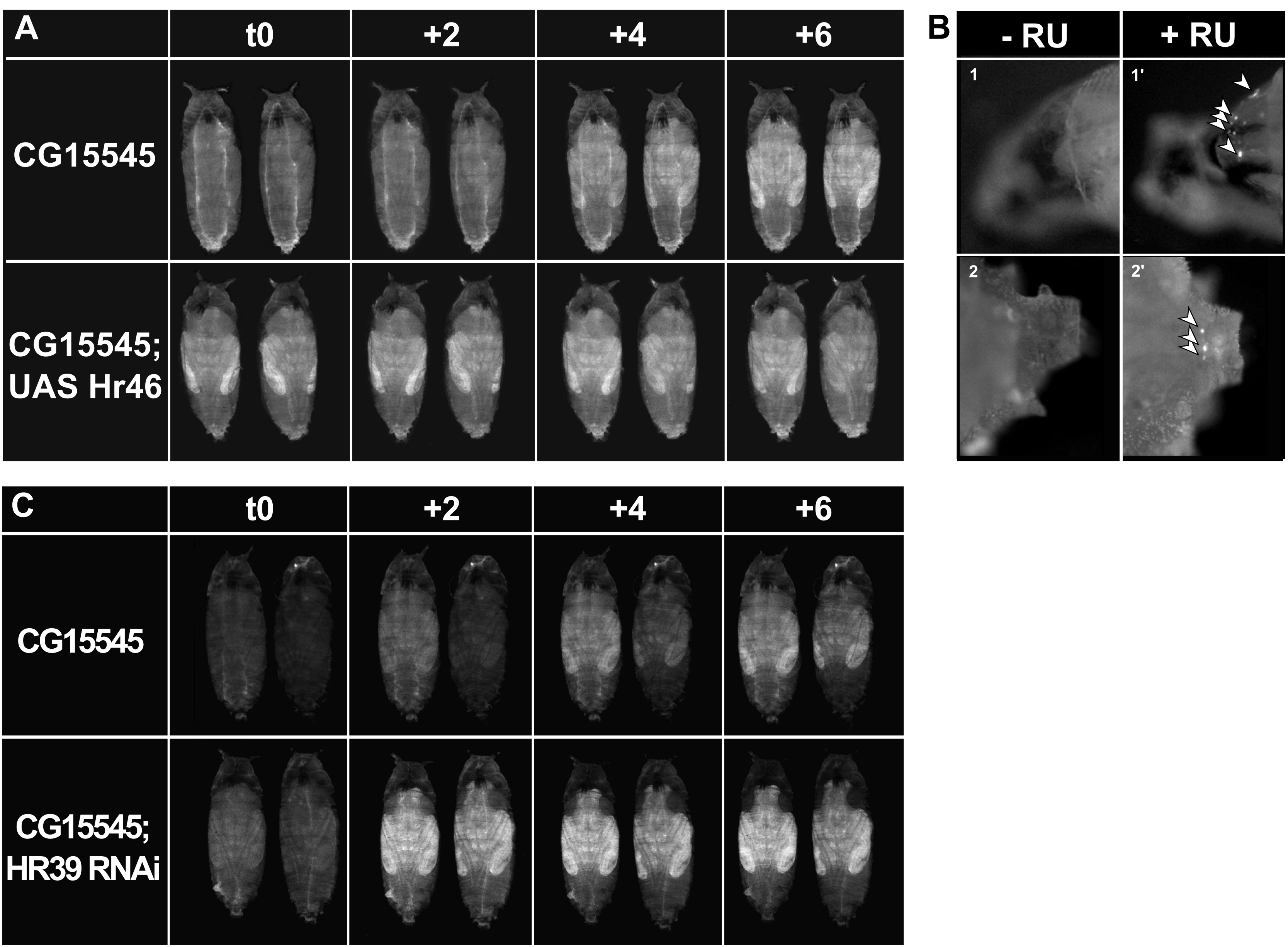

Supplement: Supplementary file 1 — Additional file 1: Table S1: CisTargetX analysis of cluster 12 gene set with 2.5 Z score cutoff. Table S2. Detailed genomic coordinates of tested CRMs. Table S3. List of primer pairs used for Q-RT PCR analysis. Figure S1. Logos of PWM outlined in Figure 1. Figure S2. Details of tested CRM.Figure S3. qRT-PCR in developing pupal wings of CG15545-GFP individuals. Figure S4. Sequence based comparison of NR motifs. Figure S5. Hr46 and Hr39 regulate CG15547-GFP expression. (DOCX 6 MB) [file 12864_2014_6234_MOESM1_ESM.docx]
